# Combined Trabectedin and anti-PD1 antibody produces a synergistic antitumor effect in a murine model of ovarian cancer

**DOI:** 10.1186/s12967-015-0613-y

**Published:** 2015-07-29

**Authors:** Zhiqiang Guo, Haolin Wang, Fandong Meng, Jie Li, Shulan Zhang

**Affiliations:** Department of Gynecology and Obstetrics, Shengjing Hospital, China Medical University, Shenyang, 110004 China; Department of Acute Abdominal Surgery, The First Hospital of Dalian Medical University, Dalian, 116044 China; Molecular Oncology Department of Cancer Research Institution, The First Hospital of China Medical University, Shenyang, 110004 China

## Abstract

**Background:**

Monoclonal antibodies (mAb) that block programmed death (PD)-1 signaling pathway hold great potential as a novel cancer immunotherapy. Recent evidence suggests that combining with conventional, targeted or other immunotherapies, these mAb can induce synergistic antitumor responses. In this study, we investigated whether Trabectedin (ET-743), a novel anticancer agent currently used for treating relapsed ovarian cancer, can synergize with anti (α)-PD-1 mAb to increase antitumor activity in the murine ID8 ovarian cancer model.

**Methods:**

Mice with established peritoneal ID8 tumor were treated with either single or combined Trabectedin and α-PD-1 mAb, their overall survival was recorded; tumor-associated immune cells and immune gene expression in tumors from treated mice were analyzed by flow cytometry and quantitative RT-PCR, respectively, and antigen-specific immunity of effector CD8^+^ T cells was evaluated by ELISA and cytotoxicity assay. In addition, the effect of Trabectedin on tumoral PD-L1 expression was analyzed by both flow cytometry and immunofluorescence staining.

**Results:**

Though single treatment showed a modest antitumor effect in mice bearing 10-day-established ID8 tumor, combined Trabectedin and α-PD-1 mAb treatment induced a strong antitumor immune response, leading to a significant tumor regression with half of mice tumor-free 90 days after tumor inoculation. Mechanistic investigation revealed that combination treatment induces a systemic tumor-specific immunity with an indispensable role of both CD4^+^ and CD8^+^ T cells, and effector CD8^+^ T cells exhibited the antigen-specific cytokine secretion and cytotoxicity upon tumor antigen stimulation; additionally, combination treatment increased the IFN-γ-producing effector T cells and decreased the immunosuppressive cells in peritoneal cavity; accordingly, it enhanced the expression of Th1-associated immune-stimulating genes while reducing the transcription of regulatory/suppressive immune genes, reshaping tumor microenvironment from a immunosuppressive to a stimulatory state. Finally, in vivo Trabectedin treatment has been shown to induce IFN-γ-dependent PD-L1 expression within tumor, possibly constituting a mechanistic basis for its synergistic antitumor effect with α-PD-1 mAb therapy.

**Conclusion:**

This study provides the evidence that α-PD-1 mAb can produce a synergistic antitumor efficacy when combined with Trabectedin, a clinically available anticancer agent, supporting a direct translation of this combination strategy in clinic for the treatment of ovarian cancer.

**Electronic supplementary material:**

The online version of this article (doi:10.1186/s12967-015-0613-y) contains supplementary material, which is available to authorized users.

## Background

Ovarian carcinoma (OC) is the most lethal malignancy in women, with 22,280 new cases and 15,460 deaths estimated in the United States for 2012 [1]. The high rate of lethality from OC is primarily due to the advanced stage of disease at diagnosis. Early stage cancers can be cured in up to 90% of patients with current therapies [2], but this rate drops substantially for advanced disease with approximately 30% of patients with advanced stage OC survive 5 years after initial diagnosis [3]. The standard treatment for ovarian cancer is surgical debulking followed by platinum-taxane based chemotherapy [4]. Although most patients are responsive to chemotherapy at first, the majority of them will eventually have a relapse and die of the disease. Therefore, novel strategies are urgently needed to improve the outcomes of ovarian cancer.

Lines of evidence suggest that OC should be amenable to the immunotherapy [5]. OC cells express many tumor-associated antigens against which specific immune responses have been detected [6–10]; furthermore, endogenous anti-tumor immunity has been thought to impose a major impact on the outcomes of patients with OC as evidenced by the close correlation between patient survival and tumoral T cell infiltration in large annotated clinical samples [11]. Lastly, a uniquely advantageous feature of OC is that their primary tumors and metastases are predominantly located in the peritoneal cavity where immunotherapeutic agents can be directly administered, thus bypassing systemic application and its associated adverse effects [12]. Despite the abundant evidence supporting OC immunotherapy, clinical success with immune-based therapies for OC has generally been modest [13].

Currently, checkpoint inhibitors have shown a great potential in the treatment of multiple types of cancer [14, 15], among which antibodies blocking programmed death 1 (PD-1) and its ligand PD-L1 produced an impressively therapeutic effect in multiple malignancies [16–21]. As for their application in OC, a clinical trial including a small cohort of OC patients demonstrated that treatment with α-PD-L1 antibody induced a durable antitumor response in a minority of patients (1 partial response and 3 stable disease in 17 patients lasting for at least 24 weeks) [16]; additionally, α-CTLA4 antibody ipilimumab has also been reported to increase antitumor effect of GM-CSF-modified autologous tumor cell vaccines [22, 23]. These preliminary clinical data suggest that immune checkpoint inhibitors are an excellent option for OC treatment though a giant space for improvement exists [24]. In this regard, our previous studies conducted in the ID8, a highly clinical relevant murine ovarian cancer, demonstrated that single checkpoint inhibitor such α-PD-1 or α-TIM3 antibody was ineffective in preventing peritoneal tumor growth while combined treatment with checkpoint inhibitors and immune-stimulating antibodies produced a potently synergistic antitumor effect, leading to the regression of the established tumors [25, 26]. The significantly improved effectiveness of combined treatment with checkpoint inhibitors is also validated in other preclinical studies [27–29] as well as a recent clinical trial designed for treating melanoma [29]. Therefore, it is urgently needed to explore optimal combined strategies to improve the antitumor efficacy of checkpoint inhibitors in OC treatment, which should be best directly translatable in clinic.

Trabectedin (ET-743) is a synthetic, marine-derived anticancer agent that binds to the minor groove of DNA, bending the helix to the major groove, resulting in perturbation of the cell cycle [30]. Trabectedin, in combination with pegylated liposomal doxorubicin hydrochloride (PLDH), has been approved by Food and Drug Administration (FDA) and Europe Medicine Agency (EMA) for the treatment of patients with relapsed platinum-sensitive ovarian cancer [31, 32]. Recent studies demonstrate the dual function of Trabectedin as it can not only directly kill tumor cells by interfering with cell cycle progression but also modulates tumor microenvironment via selective depletion of protumor monocytes, such as tumor-associated macrophages (TAM) and myeloid-derived suppressor cells (MDSC) [33]. Based on the tumor-killing and immune-modulating activity of Trabectedin, one would speculate that combined treatment of Trabectedin and checkpoint inhibitors should produce an additive and even synergistic antitumor effect in OC.

The present investigation was designed to explore the antitumor effect of combined Trabectedin and α-PD-1 antibody in ID8 murine OC model. We further characterized the cellular and molecular mechanisms driving this combined antitumor effect.

## Methods

### Mice

Female C57BL (6–8 week old) were purchased from the Animal Experimental Center of the China Medical University. Animal use was approved by our institution (China Medical University).

### Cell lines

Murine ID8 ovarian cancer cells, TC-1 lung carcinoma cells and T cell lymphoma EL4 cells were all kept in our lab as previously described [25, 26]. Tumor cells were cultured in the complete DMEM medium supplemented with 10% FBS (Thermo Scientific, Rockford, IL), 100 U/mL penicillin and 100 μg/mL streptomycin before cell suspensions were prepared and transplanted to mice. The EL4 cells and splenocytes were maintained in a complete medium of RPMI-1640 supplemented with 10% FBS, 25 mM HEPES, 2 mM glutamine, 100 U/mL penicillin and 100 μg/mL streptomycin.

### Reagents

Trabectedin was purchased from PharmaMar (Colmenar Viejo, Madrid, Spain) and dissolved in DMSO to 1 mM (761.8 µg/mL) and kept at −20°C. Therapeutic α-PD-1 (Clone RMT3-23; Catalog#BE0115), α-CD4 (Clone GK1.5; Catalog#:BE0003-1), α-CD8 (Clone 2.43; Catalog#:BE0061), α-NK1.1 (Clone PK136; Catalog#:BE0036), and control (Clone 2A3; Catalog#:BE0089) mAb were purchased from BioXcell (West Lebanon, NH). Antibodies used for flow cytometry were purchased from Tianjing Sungene (Tianjing, China) and eBioscience (San Diego, CA).

### Tumor challenge and treatment experiments

**Fig. 1:**
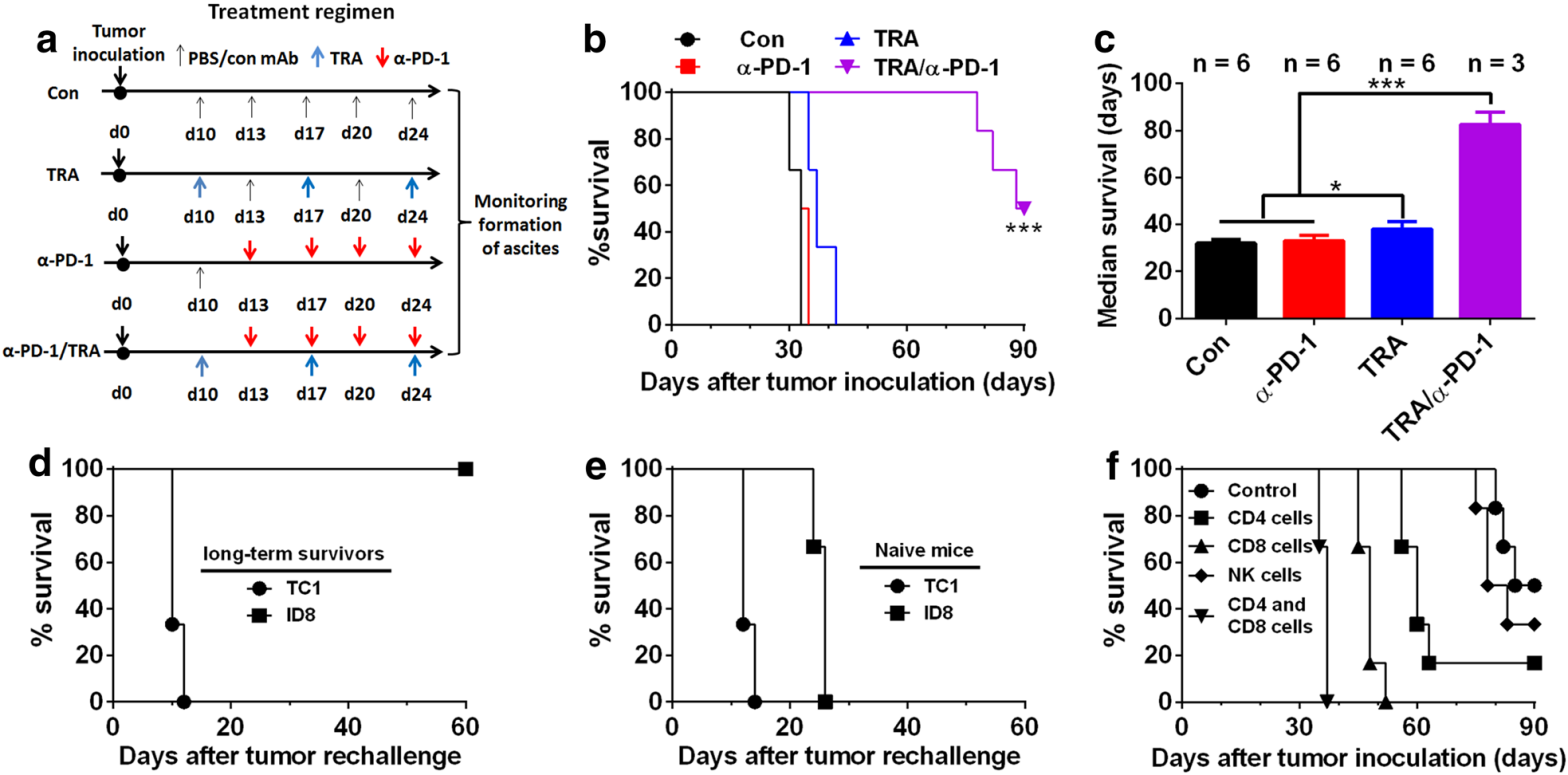
Combined treatment with Trabectedin and α-PD-1 mAb mounted a durable tumor-specific immunity. **a** Schematic regimen for combination treatment in ID8 tumor model. **b** Mice (6 mice per group) were transplanted i.p. with 1 × 10^6^ ID8 cells on day 0 and treated with either single or combined Trabectedin and α-PD-1 mAb as indicated in **a**, and overall survival of mice was recorded. **c** Median survival time of tumor-bearing mice was calculated. n indicates the number of tumor-bearing mice calculated. **d** Long-term surviving (90 days after first tumor challenge) mice from combination group (3 mice per group) were s.c. rechallenged with ID8 or unrelated TC1 tumor cells and their overall survival was recorded. **e** Naïve mice challenged with ID8 or TC1 tumor cells was used as control. **f** Mice (6 mice per group) receiving combination treatment were also injected with an α-CD4, α-CD8, α-CD4 plus α-CD8, α-NK1.1 or control mAb with 500 μg of each mAb per mouse 1 day before and 2 days after tumor challenge followed by injection of 200 μg every 5 days thereafter for the duration of the experiments and their overall survival were recorded. Data are representative of two (**f**) or three (**b**, **c**) independent experiments. *P < 0.05, ***P < 0.001.

In the experiments with ID8 ovarian tumor, mice (6 mice per group) were injected intraperitoneally (i.p.) with 1 × 10^6^ ID8 cells in 0.1 mL of PBS. Starting from 10 days post-tumor injection, mice were treated with Trabectedin and/or α-PD-1 mAb as scheduled in Fig. 1a. Trabectedin was administered intravenously at a dose of 200 µg/kg (body weight) once per week for 3 weeks while α-PD-1 mAb was peritoneally injected twice per week for 2 weeks. Control mice received the injection of PBS. The mice were weighted twice weekly and checked daily for the clinical sign of swollen bellies indicative of ascites information and for the evidence of toxicity such as respiratory distress, mobility, weight loss, diarrhea, hunched posture, and failure to eat while histopathology was conducted on major organs (i.e., liver, kidney, intestines, lungs, and colon). Following institutional guidelines, mice were killed when they developed ascites and had a weight increase >30%. The survival of each mouse was recorded and overall survival was calculated.

For assessing the development of immune memory, pooled (2 independent experiment) 6 long-term surviving mice (90 days after first tumor injection) from first combination treatment or age-matched naïve mice (which served as control) were challenged subcutaneously (s.c.) with 1 × 10^6^ ID8 cells or 1 × 10^6^ syngeneic but antigenically different TC1 cells. Three perpendicular diameters of s.c. tumors were measured every second day using a caliper and tumor volumes were calculated according to the formula: 1/2 × (length) × (width)^2^. Mice were sacrificed when they seemed moribund or their tumors reached 10 mm in diameter.

For depletion of immune cells, mice were injected i.p. with 500 μg of mAbs to CD8, CD4, CD4 plus CD8, or NK1.1, 1 day before and 2 days after tumor challenge, followed by injection of 250 μg every 5 days throughout the experiment. The efficacy of cell depletion was verified by staining peripheral blood leukocytes for specific subsets after depletion (data not shown).

### Evaluation of tumor-associated immune cells (TIC)

Analysis of TIC composition was performed as previously described [25, 26]. Briefly, TIC were prepared from peritoneal lavage of treated mice 7 days after commencement of treatment by using a mouse lymphocyte isolation buffer (Cedarlane, Burlington, Ontario) following the manufacturer’s instruction. TIC were then washed with FACS staining buffer and incubated with mouse Fc receptor binding inhibitor (eBioscience) for 10 min before staining with mAbs (Tianjing Sungene) against mouse CD45 (clone 30-F11), CD3 (clone 145-2C11), CD4 (clone GK1.5), CD8 (clone 53-6.7), CD11b (clone M1/70) and Gr-1 (clone RB6-8C5) for 30 min. For intracellular staining of FoxP3 (clone FJK-16s; eBioscience), cells were fixed, permeabilized and stained following the instruction of Cytofix/Cytoperm kit (BD Bioscience). For intracellular staining of IFN-γ (clone XMG1.2), cells were restimulated in vitro with 50 ng/mL PMA and 1 μg/mL ionomycin for 4 h prior to the analysis of IFN-γ secretion in CD4^+^ or CD8^+^ T cells. Flow cytometry was performed using FACSCalibur (BD Biosciences) and the lymphocyte population was selected by gating CD45-positive cells. The data were analyzed using Flow Jo software (Tree Star, Ashland, OR, USA). All flow cytometry experiments were performed at least two times.

### Quantitative RT-PCR

Quantitative RT-PCR was performed as previously described [25, 26]. Briefly, tumors were excised from treated mice 7 days after treatment commencement from which total RNA was extracted using RNeasy Mini Kits (Qiagen, Hilden, GA, USA) and reverse transcribed into cDNA using SuperScript III Reverse Transcriptase (Invitrogen). Expression for genes of interest was analyzed in tumors harvested 7 days after tumor commence. The primers for all genes tested, including internal control GAPDH, were synthesized by Takara Inc., Dalian, China. Primer sequences were listed in Additional file 1: Table S1. Quantitative real-time PCR was performed via ABI PRISM 7500 Real-Time PCR Systerm (Applied Biosystems) with 1 × SYBR Green Universal PCR Mastermix (Takara). Transcript levels were calculated according to the 2^−ΔΔCt^ method, normalized to the expression of GAPDH, and expressed as fold change compared with control.

### Evaluation of antigen-specific CTL immune response

Isolated splenocytes from treated mice were cultured in the presence of 10 μg/mL H-2Db-restricted mesothelin-derived peptides (amino acid 406-414) or control HPV-E7-derived peptide (amino acid 49-57; all from GenScript, Nanjing, CA, USA) for 3 days. IFN-γ in the supernatants was determined by Mouse IFN-γ Quantikine ELISA Kit (R&D systems, Minneapolis, MN, USA).

For CTL assays, effector cells were obtained by coculturing 5 × 10^6^ splenocytes with 5 × 10^5^ UV-irradiated ID8 cells for 4 days. Peptide-pulsed EL4 target cells were generated by adding 10 μg/mL of peptide and incubating for 4 h. CTL activity was measured using the CytoTox96 Non-Radioactive Cytotoxicity Assay kit (Promega, Madison, WI, USA) following the manufacturer’s instructions. In brief, target cells were incubated with varying numbers of effector cells for about 4 h, and supernatants were then analyzed for lactate dehydrogenase release. The results are expressed as percent specific lysis, calculated as (Experimental release-Spontaneous release/Total release-Spontaneous release) × 100. In some experiments, effector cells were incubated with anti-CD4 or CD8 antibody (10 μg/mL) for 2 h before CTL assay.

### Evaluation of PD-L1 within tumors

For PD-1 measurement by flow cytometry, B6 mice were inoculated i.p. with 1 × 10^6^ ID8 cells and treated with Trabectedin on day 10 and 17. On day 19, tumor masses harvested from mice were cut into small pieces, disaggregated with collagenase (0.5 mg/mL), and filtered through strainers. Cells (10^6^) were stained with APC conjugated anti-PD-L1 (clone 10F.9G2) or isotype control (clone eBR2a; rat IgG2a) mAb (all from Biolegend), and PD-L1 expression was analyzed by flow cytometry as above. In addition, ID8 tumor cells (5 × 10^5^) cultured in 6-well plates were treated with peritoneal lavage fluid (20%, v/v) prepared from control or Trabectedin treated mice for 24 h and PD-L1 expression was then analyzed by flow cytometry. In some experiments, ID8 tumor cells treated with peritoneal lavage fluid from Trabectedin treated mice were cultured in the presence of isotype (clone RTK2071; rat IgG1) or anti-IFN-γ neutralizing antibody (clone AN-18; all from Biolegend).

For immunofluorescence staining, tumor masses harvested from treated mice were fixed with paraformaldehyde and blocked with 5% bovine serum albumin (BSA) for 45 min at room temperature. The samples were incubated with anti-mouse PD-L1 (ab80276 from Abcam; 1:100 in PBS + 5% BSA), anti-mouse CD68 (ab53444 from Abcam; 1:50 in PBS + 5% BSA) or isotype control (ab18446 from Abcam) primary antibody at 4°C for 16 h. An Alexa Fluor^®^ 488-conjugated goat anti-rat IgG (H + L) polyclonal antibody (ab150157 from Abcam; 1:200) was used as the secondary antibody. The samples were mounted in ProLong Gold anti-fade reagent (Life Technologies) and imaged on the Olympus FV1000 confocal microscope system.

### Statistics

Statistical analyses of all other data were performed by GraphPad Prism (Version 5.04, GraphPad Software, Inc). Results are presented as mean ± SEM obtained from at least two independent experiments. Differences between groups were tested by one-way or two-way ANOVA followed by Tukey’s multiple comparisons test; Survival rates were analyzed using the Kaplan–Meier method and evaluated with the log-rank test with Bonferroni correction. Significant differences were accepted at p < 0.05.

## Results

### Combined Trabectedin and α-PD-1 mAb treatment synergistically induces a durable antitumor effect depending on both CD4^+^ and CD8^+^ T cells

As Trabectedin induces a profound growth inhibition and cell apoptosis in human ovarian cancer cell lines and our previous data showed that ID8 murine ovarian cancer cells do not express PD-1 and its ligand PD-L1 on their surface [25], we first evaluated the direct effect of Trabectedin and/or α-PD-1 mAb on the survival of ID8 cells in vitro. As expected, only Trabectedin inhibited ID8 cell growth, whereas α-PD-1 mAb could neither inhibit ID8 cell growth directly nor enhance the inhibitory effect of Trabectedin (Additional file 2: Figure S1).

We tested the antitumor efficacy, defined as prolonged overall survival, of single or combined Trabectedin and α-PD-1 mAb in C57BL/6 mice transplanted i.p. 10 days previously with 1 × 10^6^ ID8 cells. Detailed treatment regimen was shown in Fig. 1a. As described in our previous studies [25], single treatment with α-PD-1 mAb showed little antitumor effect, leading to ascites formation at the almost same time as control treated mice (about 30 days post-injection). Treatment with Trabectedin alone show a modest tumor-suppressing activity, resulting in a slight increase in overall survival (Fig. 1b); however, when tumor-bearing mice received the treatment of combined Trabectedin and α-PD-1 mAb, their overall survival were significantly prolonged with 50% (3 out of 6 mice) of mice surviving at the end of experiment (90 days after tumor injection), these long-term surviving mice were tumor free in peritoneal cavity when they were examined at postmortem; though remaining half of mice succumbed to tumor growth, their median survival time was markedly longer than mice receiving control or single treatment (Fig. 1c; median survival 33, 34, 37 and 89 days for control, α-PD-1, Trabectedin and Trabectedin/α-PD-1). Two repeats of the experiment gave similar results (data not shown). We did not observe any obvious toxicity such as weight or hair loss in mice receiving combination treatment.

Notably, those long-term survivors developed the systemic tumor-specific memory immune response in that they were resistant to the rechallenge by s.c. injection of ID8 ovarian cancer cells but not s.c. injection of unrelated TC1 lung cancer cells (Fig. 1d) while naïve mice succumbed to them (Fig. 1e). Ninety days after rechallenge, 100% (3 out of 3 mice) of mice remained tumor-free. Antibody-mediated cell depletion experiments demonstrated that protection conferred by Trabectedin/α-PD-1 treatment was dependent on the CD4^+^ and CD8^+^ T cells as the antitumor effect of this combination treatment was heavily compromised in the absence of CD4^+^ or CD8^+^ T cells (Fig. 1f); NK cells play a minor role since removal of these cells slightly decreased the tumor protection.

### Combined Trabectedin and α-PD-1 mAb treatment increases ratios of effector T cells to immunosuppressive cells in peritoneal lavage

**Fig. 2:**
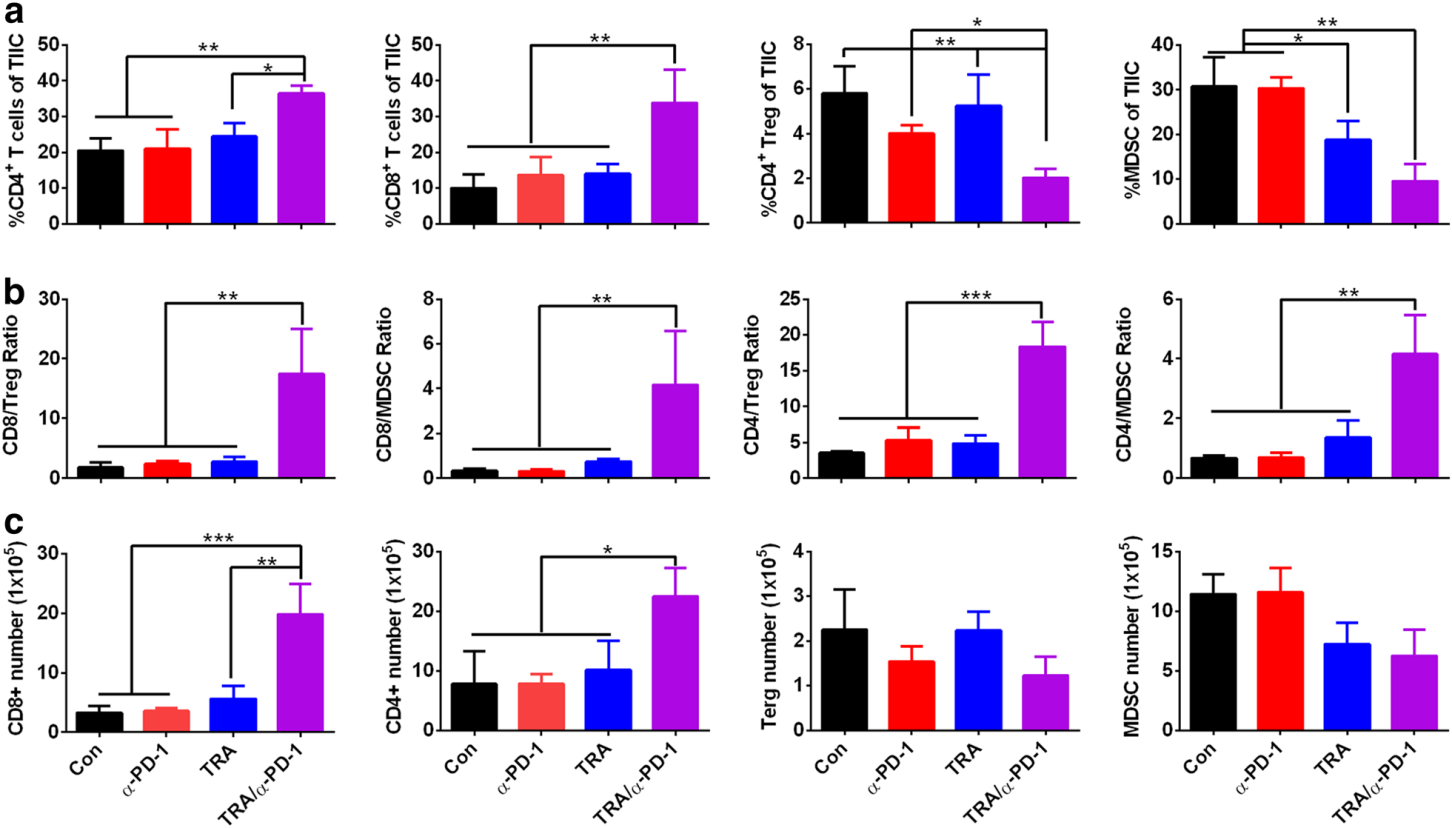
Analysis of tumor-associated immune cells in peritoneal lavage from treated mice. Mice (6 mice per group) injected i.p. with 1 × 10^6^ ID8 cells 10 day earlier were treated as described in Fig. 1a. Seven days after treatment commencement, peritoneal lavage from treated mice was analyzed by flow cytometry for the percentages and numbers of various subsets of TIC. The percentages and absolute numbers of CD4^+^FoxP3^−^, CD8^+^, CD4^+^FoxP3^+^ Treg and CD11b^+^GR-1^+^ MDSC in peritoneal lavage are shown in **a**, **c**, respectively. The ratios of CD4^+^FoxP3^−^ andvCD8^+^ T cells to Treg and MDSC in peritoneal lavage are shown in **b**. Data are representative of two independent experiments. *P < 0.05, **P < 0.01, ***P < 0.001.

To understand the synergistic mechanism of Trabectedin and PD-1 blockade in the ID8 tumor model, we evaluated the composition of tumor-associated immune cells (TIC) in peritoneal lavage of treated mice 7 days after treatment commence, focusing on antitumor effector CD4^+^FoxP3^-^ and CD8^+^ T cells and immunosuppressive CD11b^+^GR-1^+^ MDSC and CD4^+^FoxP3^+^ regulatory T cells (Treg). Compared with control treatment, α-PD-1 mAb treatment alone showed a modest effect on the composition of TIC with a slight decrease in immunosuppressive Treg which is consistent with our previous report (Fig. 2a; Ref [25]); though executing little effect on other immune subsets, single Trabectedin treatment significantly decreased the percentage of MDSC as previously described [33]; strikingly, combined Trabectedin and α-PD-1 mAb treatment significantly increased the percentage of effector CD4^+^FoxP3^-^ T cells (mean value for Trabectedin/α-PD-1 vs control, α-PD-1 and Trabectedin: 36.43 vs 20.53, 20.97 and 24.43%; p < 0.05) and CD8^+^ T cells (33.83 vs 10.03, 13.60 and 14.07%; p < 0.01) while concomitantly diminishing that of immunosuppressive Treg (2.03 vs 5.80, 4.00 and 5.23%; p < 0.05) and MDSC (9.57 vs 30.77, 30.30, 18.80%; p < 0.05), resulting in a prominent elevation in the ratios of both effector CD4^+^FoxP3^−^ and CD8^+^ T cells to immunosuppressive MDSC and Treg (Fig. 2b; CD4^+^FoxP3^−^/Treg: 18.37 vs 3.56, 5.35 and 4.85, p < 0.001; CD8^+^/Treg: 17.44 vs 1.81, 2.40 and 2.80, p < 0.01; CD4^+^FoxP3^−^/MDSC: 4.16 vs 0.68, 0.69 and 1.38, p < 0.01; CD8^+^/MDSC: 4.17 vs 0.32, 0.32 and 0.76, p < 0.01). Regarding the absolute number of TIC, we observed a similar trend with a prominent increase in effector T cells and a decrease in immunosuppressive cells from combination treatment (Fig. 2c).

**Fig. 3:**
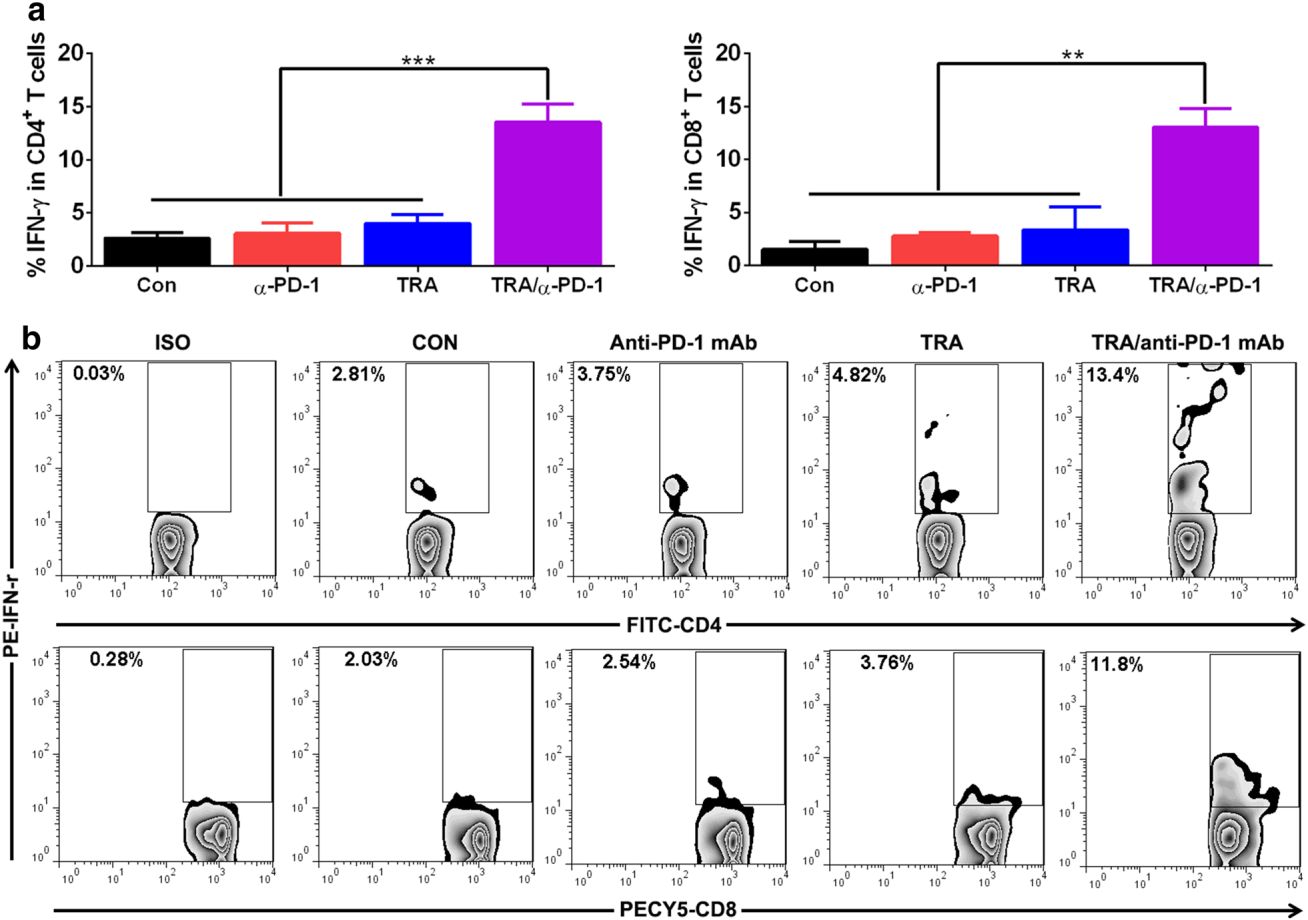
Functional analysis of IFN-γ production in peritoneal tumor-associated CD4^+^ and CD8^+^ T cells from treated mice. Mice (6 mice per group) inoculated i.p. with 1 × 10^6^ ID8 cells 10 day earlier were treated as described in Fig. 1a. Seven days after treatment commencement, tumor-associated CD4^+^ and CD8^+^ T cells from peritoneal cavity of treated mice were assayed of IFN-γ production by intracellular cytokine staining. The frequencies of IFN-γ-producing cells in tumor-associated CD4^+^ and CD8^+^ T cells are shown in **a**. The representative dotplots are shown in **b** with *upper* and *bottom panels* displaying IFN-γ staining in gated CD4^+^ and CD8^+^ T cells respectively. ISO designates the isotype staining. Data are representative of two independent experiments, **P < 0.01, ***P < 0.001.

Further functional analysis showed that significantly elevated frequencies of IFN-γ-producing cells were seen in tumor-associated CD4^+^ and CD8^+^ T cells from combination-treated mice (Fig. 3a). The representative dotplots were shown in Fig. 3b.

Together, these data indicate that combined treatment with Trabectedin and PD-1 blockade synergistically creates higher ratios of effector T cells to immunosuppressive cells in peritoneal cavity of treated mice, representing the shift of an immunosuppressive tumor milieu to an immunostimulatory state which is more permissive for immune mediated tumor destruction.

### Combined Trabectedin and α-PD-1 mAb treatment shaped a local immunostimulatory microenvironment

**Fig. 4:**
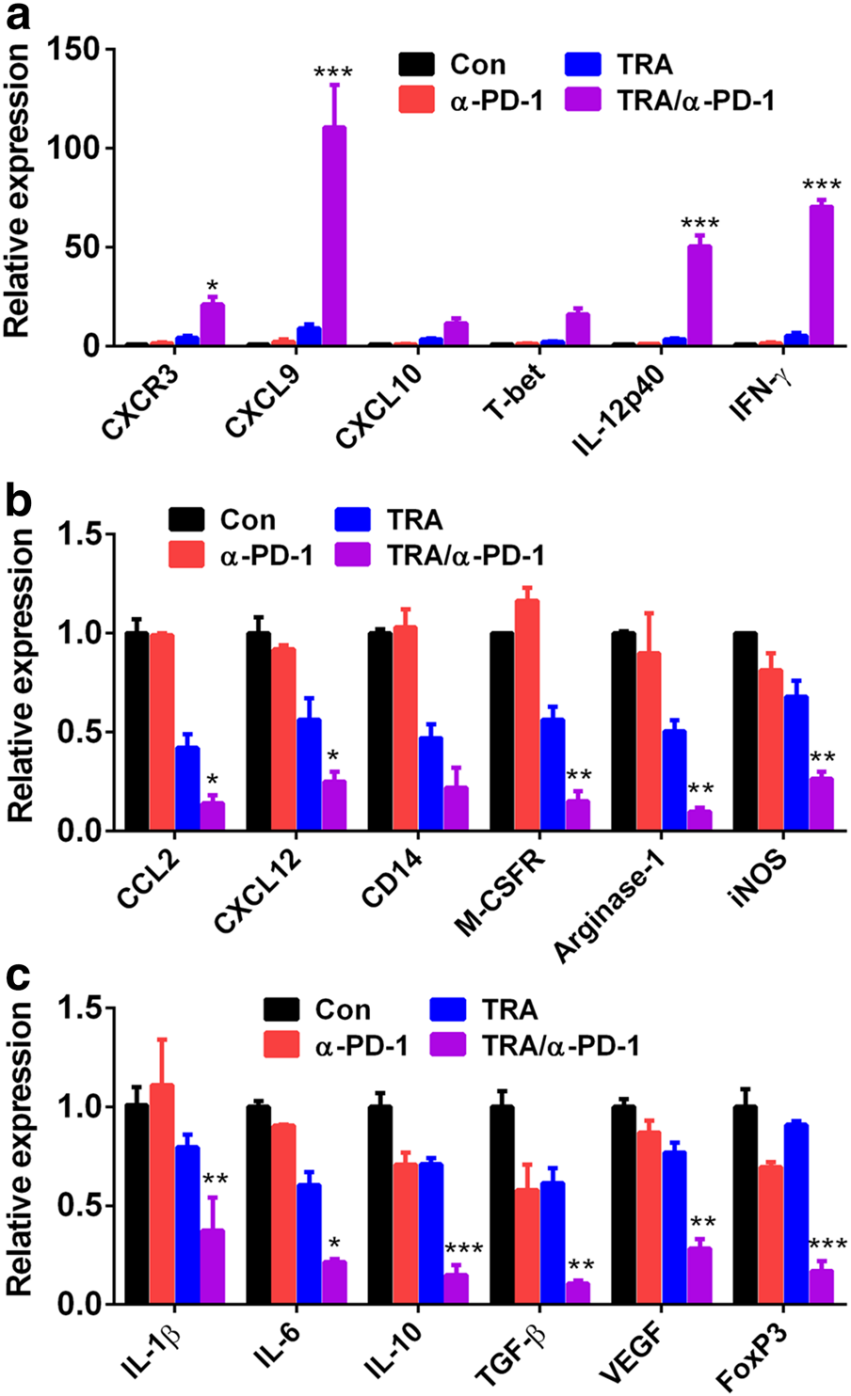
Expression profiling of immune-associated genes in tumors from treated mice. **a** Mice (6 mice per group) inoculated i.p. with 1 × 10^6^ ID8 cells 10 day earlier were treated as described in Fig. 1a. Seven days after treatment commencement, tumors were excised and their RNA were extracted for the analysis of immune-associated gene expression by quantitative RT-PCR. **a** The expression of Th1-related molecules CXCR3, CXCL9, CXCL10, T-bet, IL-12p40 and IFN-γ. **b**, **c** The expression of genes relating with TAM/MDSC and Treg recruitment/accumuation and function, including CCL2, CXCL12, CD14, M-CSFR, Arginase-1, iNOS, IL-1β, IL-6, IL-10, TGF-β, VEGF and FoxP3. *P < 0.05, **P < 0.01, ***P < 0.001.

To deepen insight into the experienced change of tumor immune microenvironment induced by combination treatment, we performed expression profiling of 20 immune-associated genes in tumors by using quantitative RT-PCR. As shown in Fig. 4a, combined Trabectedin/α-PD-1 mAb treatment induced a marked change of immune-associated genes marked by increased Th1 effector T cell recruitment (i.e., increased transcript expression for CXCR3 and its ligands CXCL9 and CXCL10) and functionality (i.e., increased transcripts for IFN-γ, IL-12p40 and T-bet). The tumor treated with Trabectedin/α-PD-1 mAb also exhibited the decreased protumor TAM/MDSC and Treg recruitment/accumulation and (i.e., reduced transcript expression for CCL2, CXCL12, CD14, M-CSFR and FoxP3; Fig. 4b) and function (i.e., decreased expression of transcripts for IL-1β, IL-6, IL-10, TGF-β and VEGF as well as the MDSC-associated gene products arginase-1 and iNOS; Fig. 4c). Consistent with the above-mentioned analysis of TIC composition, treatment with Trabectedin alone decreased the expression of TAM/MDSC-associated marker (i.e., reduced transcripts for CCL2, CXCL12, CD14 and M-CSFR), function (i.e., reduced transcripts for arginase-1 and iNOS) and inflammatory cytokine (i.e., reduced transcripts for IL-1β and IL-6) genes while single α-PD-1 treatment attenuated the transcription of genes with Treg (i.e., reduced expression for IL-10, TGF-β and FoxP3). The depleting effect of Trabectedin on TAM was further confirmed by immunofluorescence staining where the number of CD68-postive TAM within tumors from mice treated with Trabectedin or Trabectedin/α-PD-1 mAb significantly decreased compared with that from mice treated with control or singleα-PD-1 mAb (Additional file 3: Figure S2). In sum, these results corroborate the above observation of the switch from an immunosuppressive to a predominantly antitumor Th1/CTL response within tumors from combination treated mice.

### Combined Trabectedin and α-PD-1 mAb treatment mounted an antigen-specific CTL response

**Fig. 5:**
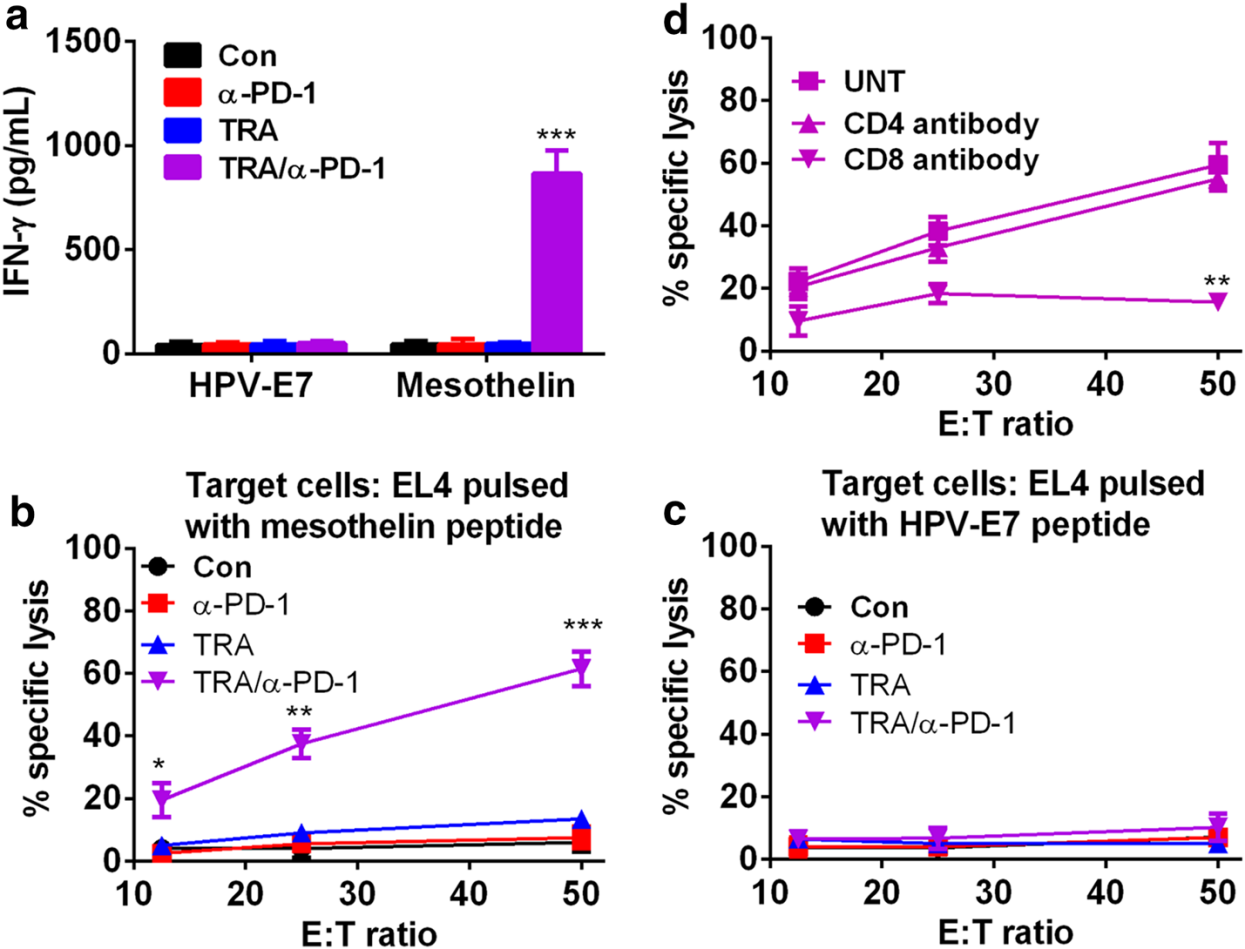
Mice treated with combined Trabectedin and α-PD-1 mAb developed a tumor antigen-specific CTL response. **a** Mice (6 mice per group) inoculated i.p. with 1 × 10^6^ ID8 cells 10 day earlier were treated as described in Fig. 1a. Two weeks after treatment commencement, splenocytes from treated mice were cultured in the presence or absence of H-2Db-restricted mesothelin-derived peptides or control HPV-E7-derived peptide for 3 days and IFN-γ production in the supernatants were assayed by ELISA. **b**, **c** Splenocytes (5 × 10^6^) from treated mice were incubated with 5 × 10^5^ UV-irradiated ID8 cells for 4 days prior to subjected to analysis of antigen-specific CTL activity by CytoTox 96 Non-radioactive cytotoxicity assay using EL4 cells pulsed with H-2Db-restricted mesothelin (**b**) or HPV-E7 peptide (**c**) as target cells. **d** The killing activity of restimulated splenocytes from combination-treated mice was also evaluated in the presence of α-CD4, α-CD8 or control antibody. Data are expressed as M ± SEM of triplicate wells. *P < 0.05, **P < 0.01, ***P < 0.001.

We next dissected the antigen-specific antitumor immune response by analyzing the splenocytes from treated mice stimulated with epitope peptide derived from mesothelin, a well-characterized tumor antigen expressed by ID8 tumor cells as previously described [25, 26]. As shown in Fig. 5a, splenocytes from combination-treated mice produced a significantly increased IFN-γ when stimulated with the H-2Db-restricted mesothelin-specific peptide as compared with that from mice of control or single treatment. We did not detected the increased secretion of IFN-γ in cultured splenocytes from all treated mice when they were stimulated with control HPV-E7 peptide, demonstrating that combination-treated mice mounted mesothelin-specific immune response. Further analysis of antigen-specific cytotoxicity showed that splenocytes from combination-treated mice exhibited a significant higher level of cytotoxic activity against EL4 cell pulsed with mesothelin but not with control HPV-E7 peptide (Fig. 5b, c). No cytotoxicity against either mesothelin or HPV-E7 peptide pulsed EL4 cells was detected from the splenocytes from mice receiving control or single treatment. Moreover, preincubation with CD8 antibody suppressed the cytolytic activity of splenocytes from combination-treated mice (Fig. 5d), suggesting that CD8^+^ T cells mediated the tumor antigen-specific killing effect.

### In vivo Trabectedin treatment induced PD-L1 expression within ID8 tumors

**Fig. 6:**
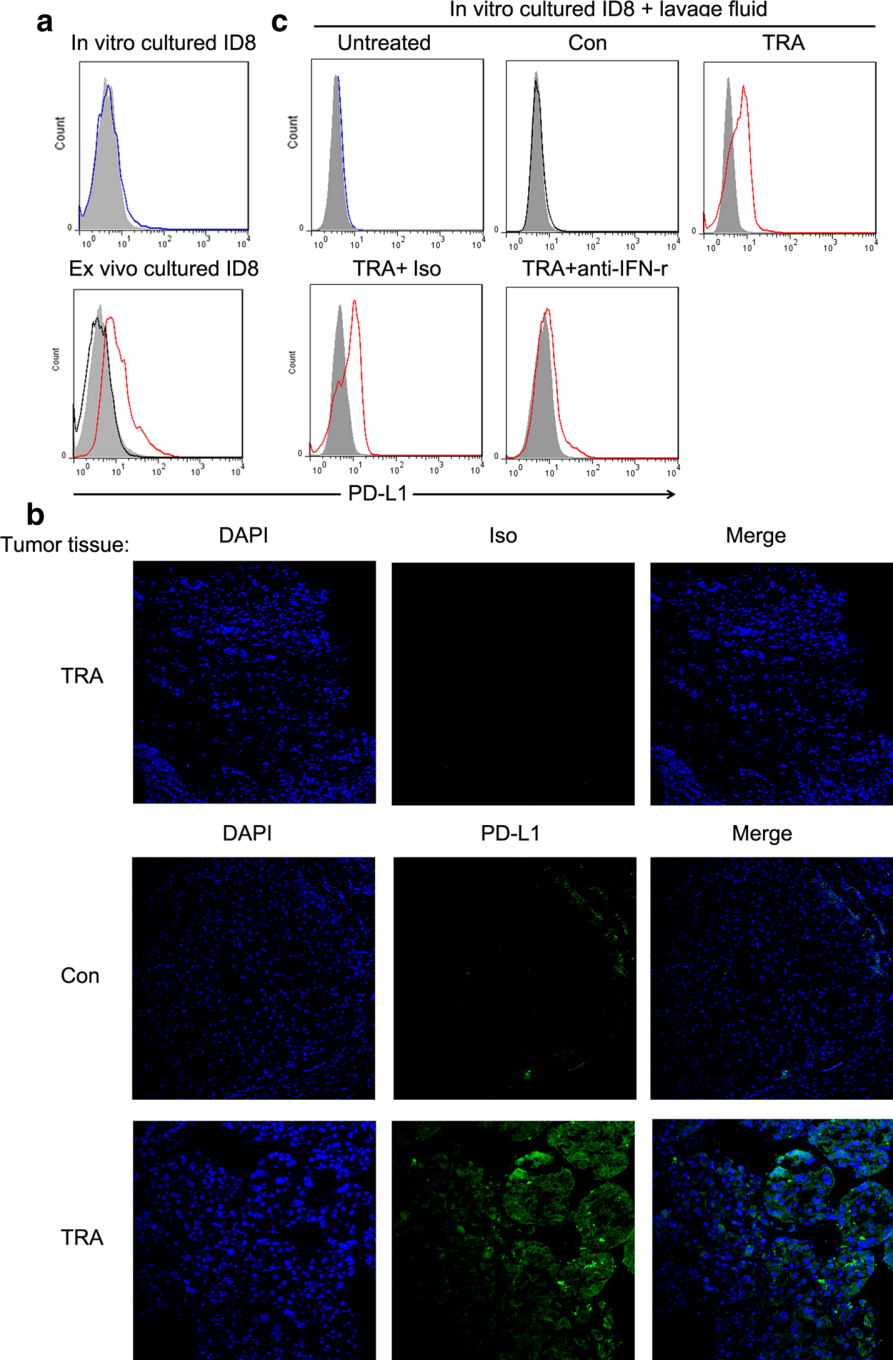
In vivo Trabectedin treatment induced PD-L1 expression within ID8 tumors. **a** Mice were inoculated i.p. with 1 × 10^6^ ID8 cells and treated with control or Trabectedin on day 10 and 17. On day 19, harvested tumor cells from treated mice were stained with APC conjugated anti-PD-L1 (*black* or *red line* for control or Trabectedin treatment) or isotype control antibody (*filled gray line*) for evaluating PD-L1 expression by flow cytometry. In vitro cultured ID8 tumor cells were included as a control (*blue line*). **b** Mice were treated as described in **a**, and harvested tumor tissues were analyzed for PD-L1 expression by immunofluorescence staining. Iso denotes isotype control staining. **c** In vitro cultured ID8 tumor cells were treated with peritoneal lavage fluid (20%, v/v) prepared from control (*black line*) or Trabectedin (*red line*) treated mice for 24 h and PD-L1 expression was then analyzed by flow cytometry (*upper panels*). In some experiments, ID8 tumor cells treated with peritoneal lavage fluid from Trabectedin treated mice were cultured in the presence of isotype or α-IFN-γ neutralizing antibody (*bottom panels*). Untreated ID8 cells were included as a control (*blue line*). Data are representative of two independent experiments.

As the presence of tumor antigen-specific preexisting T cells that are disabled by tumor PD-L1 expression is likely required for α-PD-1 mAb treatment to work [15], we speculate that Trabectedin treatment may induce tumoral PD-L1 expression by IFN-γ release from effect T cells, namely adaptive resistance mechanism recently described [34], thus providing the target for α-PD-1 mAbs. To this end, we harvested the tumor masses from mice treated with control or Trabectedin and examined the PD-L1 expression by both flow cytometry and immunofluorescence staining. Though control-treated tumors exhibited a negligible expression of PD-L1 on their surface, in vivo Trabectedin treatment induced a pronounced PD-L1 expression within tumors (Fig. 6a, b). In vitro direct treatment of ID8 with Trabectedin did not promoted the expression of PD-L1 (data not shown), indicating PD-L1 expression by in vivo Trabectedin treatment is an indirect effect. As IFN-γ is a key inducer for PD-L1 expression and single Trabectedin treatment upregulated the expression of IFN-γ (Fig. 4a), we speculate that IFN-γ release from Trabectedin treatment induced the PL-L1 expression within tumors. To this end, we treated in vitro cultured ID8 cells with lavage fluid from control or Trabectedin treated mice. As shown in Fig. 6c, lavage fluid from Trabectedin but control treated mice induced the PL-L1 expression, which can be largely prevented by the addition of IFN-γ-neutralizing but not control isotype antibody, supporting a key role of IFN-γ release in PD-L1 induction. The data indicate that in vivo Trabectedin treatment induced PD-L1 expression within tumors, which may constitute the basis for synergistic effect of combined Trabectedin/α-PD-1 mAb treatment.

## Discussion

The immunotherapeutic modalities targeting single molecule or pathway remain insufficient to achieve durable clinical responses in patients with advanced OC [35], which is simply exemplified by the fact that single treatment with even “brilliant star” antibodies targeting immune checkpoint PD-1 did not produce an optimal antitumor response in OC patients [17], accordingly, studies from us and other groups found that single treatment with α-PD-1 mAb showed little antitumor effect in mice bearing ID8 tumor [25–29]. These preclinical experiments and clinical practices as well as data from other tumors point to the importance of exploring the combination immunotherapy with rationale mechanisms [15, 36], in this regard, our previous studies demonstrate that concomitant checkpoint blockade and costimulatory molecule triggering, such as combined α-PD-1/α-OX40 or α-TIM3/CD137 mAb, produced a highly synergistic antitumor efficacy in ID8 tumor [25, 26]. However, except of α-PD-1 mAb, all other mAbs are in the early development [37], therefore, those promising strategies are currently impossible to be translated into clinic. To this end, we evaluated the synergistic antitumor efficacy of α-PD-1 mAb combined with Trabectedin, an anticancer agent with new mechanisms having been approved for the treatment of relapsed OC.

In this study, we demonstrate that combined Trabectedin/α-PD-1 mAb significantly inhibited the peritoneal tumor formation of ID8 ovarian cancer cells injected 10 days prior to treatment, resulting in the long-lasting survival of 50% of mice, whereas either treatment alone was ineffective in tumor protection. More importantly, long-term survivors from combination-treated group are able to reject the rechallenge of the same tumors as first inoculation, but not unrelated tumors, suggesting the development of tumor-specific memory immunity in these mice. The findings support that combined Trabectedin/α-PD-1 mAb treatment can mount a potently durable antitumor response, which may serve as a newly translatable immunotherapeutic option for the treatment of ovarian cancer.

We initially explored the cellular effector mechanisms by which combined treatment mediates a potent antitumor effect of. Cell depletion experiments pinpoint to a pivotal role of both CD4^+^ and CD8^+^ effector T cells in tumor protection conferred by combination treatment since removal of each subset significantly attenuated the protective effect. Furthermore, analysis of TIC composition showed that combination treatment remarkably increased the accumulation of IFN-γ-producing CD4^+^ and CD8^+^ effector T cells, while concomitantly decreasing the immunosuppressive Treg and MDSC in peritoneal lavage, giving rise to the significantly increased ratios of both CD4^+^ and CD8^+^ effector T cells to the immunosuppressive cells, a long recognized determinant of productive antitumor immune response. The balance tipping toward antitumor effectors was also reflective in a prominently increased expression of Th1-associated immune genes and concomitantly decreased transcript for genes relating to the recruitment and functionality of Treg and MDSC in tumors. The data from the analysis of immune cell composition and immune gene expression in tumors support that combination treatment induces the shift of tumor immune microenvironment from the suppressive to the stimulatory state, which favorably contributes to immune-mediated tumor destruction.

Additionally, we found that combination-treated mice developed an antigen-specific CD8^+^ T-cell immune response as evidenced by mesothelin epitope-specific IFN-γ production and cytotoxicity by splenic CD8^+^ T cells, also consistent with a pivotal role of CD8^+^ T cells in mediating tumor protection in these mice. As an endogenous non-mutated antigen, mesothelin should be naturally tolerized against; the induction of mesothelin-specific CD8^+^ T-cell immunity in this study demonstrates that endogenous tolerance to mesothelin was overcome. The development of mesothelin-specific CD8^+^ T-cell immunity seems being a useful marker to predict a successful tumor protection as previous studies testing multiple immunotherapeutics in animals and humans also show a positive parallel between antitumor efficacy and mesothelin-specific CD8^+^ T-cell immunity [25, 26, 29, 38, 39]. Thus, it may be an option to evaluating tumor antigen-specific immune responses against neoantigen or even endogenous non-mutated antigens to select cancer patients who would most benefit from checkpoint blockade therapies [40, 41]. Consistent with a critical role of CD4^+^ T cells in helping the formation of CD8^+^ CTL [42], depletion of CD4^+^ T cells also attenuated the protective effects induced by combination treatment. CD4^+^ CTL has also been described [43]; however, it remains to be determined whether CD4^+^ T cells in combination treated mice exhibit a cytolytic activity against tumors. In addition, we did not detect mesothelin-specific antibodies in sera harvested 90 days after tumor challenge from combination treated mice by using flow cytometry-based approach previously described [26], but the presence of these antibodies cannot be completely excluded in view of comparatively low sensitivity of this approach and possibly suboptimal time point for sera collection. Serially collection of sera at different time points after treatment and utilization of more sensitive approaches such as ELISA should be warrant in our future work.

The mechanisms for the synergy between α-PD-1 mAb and Trabectedin remain to be fully elucidated. Our data demonstrate that in vivo Trabectedin treatment induced the PD-L1 expression within tumors, which may be partially responsible for the synergistic antitumor effect of combined Trabectedin and α-PD-1 mAb as tumor PD-L1 expression disabling the antitumor function of preexisting tumor antigen-specific T cells has been thought to be prerequisite for α-PD-1 mAb therapy to work [15]. Previous studies have demonstrated that chemotherapeutic drugs can also potentiate or induce PD-L1 expression on tumor cells by either indirect (IFN-γ-dependent) or direct (ERK signal-mediated) manner [44, 45]. Together with our results, these studies provide evidence that several chemotherapeutic drugs have the capacity to induce PD-L1 expression, which may itself mediate the chemotherapy resistance on the one hand [46], but also provide the rationale for combined cancer treatment of chemotherapeutic drugs and anti-PD-1/PD-L1 mAbs on the other hand. In fact, anti-PD-1 mAb has been reported to show acceptable toxicity profile and encouraging antitumor activity in combination with chemotherapy in patients with advanced non-small cell lung cancer [47]. Thus, future studies are warranted to evaluate the effect of other chemotherapeutic drugs on tumoral PD-L1 expression to find out the optimal matching drugs for combined cancer treatment with anti-PD-1/PD-L1 mAbs.

## Conclusion

We reported that α-PD-1 mAb can produce a potently durable antitumor immune response when combined with Trabectedin, a clinically available antitumor agent. The findings provide proof-of-concept evidence for combined Trabectedin/α-PD-1 mAb treatment of advanced OC. Certainly, more detailed regimen studies are needed to obtain an optimal therapeutic index (maximal anti-tumor efficacy and minimum side effects) using improved mouse models before translating it to clinic.

## Additional files

Additional file 1:
**Table S1.** Primers used in Real-Time PCR. Additional file 2:
**Figure S1.** In vitro inhibitory activity of Trabectedin on ID8 tumor cells. ID8 tumor cells were treated with either single or combined Trabectedin and α-PD-1 mAb at indicated doses. After 48 h of treatment, cell proliferation assays were performed using CellTiter 96 Aqueous One Solution Cell Proliferation Assay kit (Promega) according to the manufacturer’s instructions. Absorbance was measured at 490 nm using a microplate reader (Molecular devices). The percentage of cell survival was defined as the relative absorbance of untreated versus treated cells. The assays were performed in triplicate and repeated three times. Data are shown as mean ± SEM (n = 3, in triplicate). Additional file 3:
**Figure S2.** The depleting effect of Trabectedin treatment on TAM within tumors. Mice were inoculated i.p. with 1 × 10^6^ ID8 cells and treated with either single or combined Trabectedin and α-PD-1 mAb as indicated in A on day 10 and 17. On day 19, tumor tissues harvested from treated mice were fixed with paraformaldehyde and blocked with 5% bovine serum albumin (BSA) for 45 min at room temperature. The samples were incubated with rat anti-mouse CD68 or rat IgG2a isotype control primary antibody at 4 °C for 16 h. An Alexa Fluor^®^ 488-conjugated goat anti-rat IgG (H + L) polyclonal antibody was used as the secondary antibody. The samples were then analyzed for the presence of CD68-positive TAM by Olympus FV1000 confocal microscope system. The images are representative from one tumor in each treatment.

## Abbreviations

OC: ovarian carcinoma
mAb: monoclonal antibody
PD-1: programmed cell death-1
PD-L1: PD ligand 1
CTLA-4: cytotoxic lymphocyte associated antigen 4
PLDH: pegylated liposomal doxorubicin hydrochloride
FDA: Food and Drug Administration
EMA: Europe Medicine Agency
TAM: tumor-associated macrophages
Treg: regulatory T cells
MDSC: myeloid-derived suppressor cells
Th1: T-helper 1
CTL: cytotoxicity T lymphocytes
IFN-γ: interferon-gamma
IL: interleukin
DMEM: Dulbecco’s minimum essential medium
RPMI: Roswell Park Memorial Institute
NK: natural killer
DC: dendritic cells
PBS: phosphate-buffered saline
FBS: fetal bovine serum
RT-PCR: reverse transcription polymerase chain reaction
GAPDH: glyceraldehyde phosphate dehydrogenase
SEM: standard error of mean
DMSO: dimethylsulfoxide
