# Supplementary material for: Combined Trabectedin and anti-PD1 antibody produces a synergistic antitumor effect in a murine model of ovarian cancer
Source: J Transl Med. 2015 Jul 29;13:247. doi: 10.1186/s12967-015-0613-y (PMC4517526; doi:10.1186/s12967-015-0613-y)
Supplement: Additional file 1: — Table S1. Primers used in Real-Time PCR. [file 12967_2015_613_MOESM1_ESM.docx]

**Table S1. Primers used in Real-Time PCR**

| GAPDH | Sense:5′-GTGGAGATTGTTGCCATCAACG-3′  Antisense:5′-CAGTGGATGCAGGGATGATGTTCTG-3′ |
| --- | --- |
| TGF-β1 | Sense:5′-GTGGTATACTGAGACACCTTGG-3′  Antisense:5′-CCTTAGTTTGGACAGGATCTGG-3′ |
| IL-10 | Sense:5′-CTCTTACTGACTGGCATGAGG-3′  Antisense:5′-CCTTGTAGACACCTTGGTCTTGGAG-3′ |
| FoxP3 | Sense:5′-CAGCTGCCTACAGTGCCCCTAG-3′  Antisense:5′-CATTTGCCAGCAGTGGGTAG-3′ |
| IL-1β | Sense: 5′- TCAGGCAGGCAGTATCACTC-3′  Antisense: 5′-GCAGTTGTCTAATGGGAACG-3′ |
| IL-6 | Sense: 5′- ATTTCCTCTGGTCTTCTGGA-3′  Antisense: 5′- TGGATGGTCTTGGTCCTTA-3′ |
| VEGF | Sense: 5′- ATTGAGACCCTGGTGGACA-3′  Antisense: 5′-TCTGCTGTGCTGTAGGAAGC-3′ |
| CD14 | Sense: 5′- TCTCAGTTACAACAGGCTGG-3′  Antisense: 5′- TTCTCCGAGTGGGATTCA-3′ |
| M-CSFR | Sense:5′- GGCAGGCTGGAATAATCTG-3′  Antisense: 5′- CTGGGCTTCATCACACCTA-3′ |
| Arginase-1 | Sense:5′- GAAGAATGGAAGAGTCAGTGTG-3′  Antisense: 5′- TGTTGATGTCAGTGTGAGCA-3′ |
| iNOS | Sense:5′- ACAGGGAAGTCTGAAGCACTA-3′  Antisense: 5′- GTGTGGTTGAGTTCTCTAAGCA-3′ |
| CXCR3 | Sense:5′-CTGCTGCCCAGTGGGTTT-3′  Antisense: 5′-GTTGATGTTGAACAGGGCACC-3′ |
| CXCL9 | Sense:5′-TTTTGGGCATCATCTTCCTGG-3′  Antisense: 5′-GAGGTCTTTGAGGGATTTGTAGTGG-3′ |
| CXCL10 | Sense:5′-CTTCTGAAAGGTGACCAGCC-3′  Antisense: 5′-GTCGCACCTCCACATAGCTT-3′ |
| IFN-γ | Sense: 5′-AAAAACCTAAAAAATCTAAATAACT-3′  Antisense: 5′-ATCAACAACAACTCCTTTTCCACTT-3′ |
| T-bet | Sense: 5′-CGGTACCAGAGCGGCAAGT-3′  Antisense: 5′-AGCCCCCTTGTTGTTGGTG-3′ |
| CCL2 | Sense:5′- CAATGAGTAGGCTGGAGAGC-3′  Antisense: 5′- TGAAGACCTTAGGGCAGATG-3′ |
| CXCL12 | Sense:5′-CCTCAACACTCCAAACTGT -3′  Antisense: 5′-CTTTCTCTTCTTCTGTCGCTTC-3′ |
| IL-12p40 | Sense:5′-ACATCACCTGGACCTCAGAC -3′  Antisense: 5′- TTCCTTCTTGTGGAGCAGC-3′ |
