# Supplementary figures and images for: Combined Trabectedin and anti-PD1 antibody produces a synergistic antitumor effect in a murine model of ovarian cancer
Source: J Transl Med. 2015 Jul 29;13:247. doi: 10.1186/s12967-015-0613-y (PMC4517526; doi:10.1186/s12967-015-0613-y)

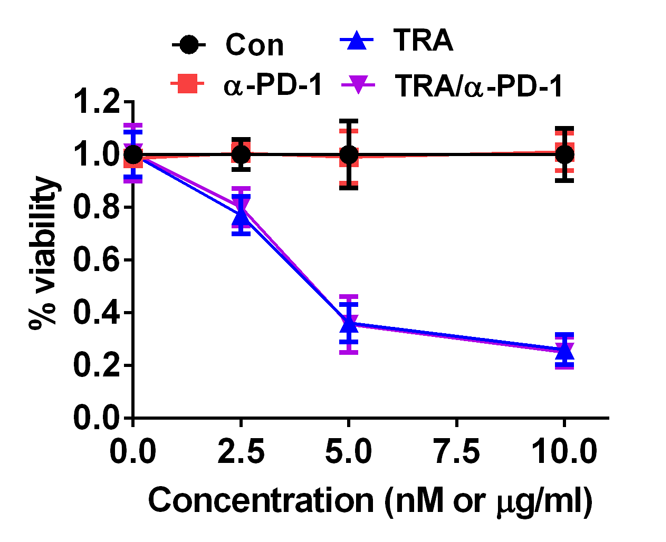

Supplement: Additional file 2: — Figure S1. In vitro inhibitory activity of Trabectedin on ID8 tumor cells. ID8 tumor cells were treated with either single or combined Trabectedin and α-PD-1 mAb at indicated doses. After 48 h of treatment, cell proliferation assays were performed using CellTiter 96 Aqueous One Solution Cell Proliferation Assay kit (Promega) according to the manufacturer’s instructions. Absorbance was measured at 490 nm using a microplate reader (Molecular devices). The percentage of cell survival was defined as the relative absorbance of untreated versus treated cells. The assays were performed in triplicate and repeated three times. Data are shown as mean ± SEM (n = 3, in triplicate). [file 12967_2015_613_MOESM2_ESM.tiff]

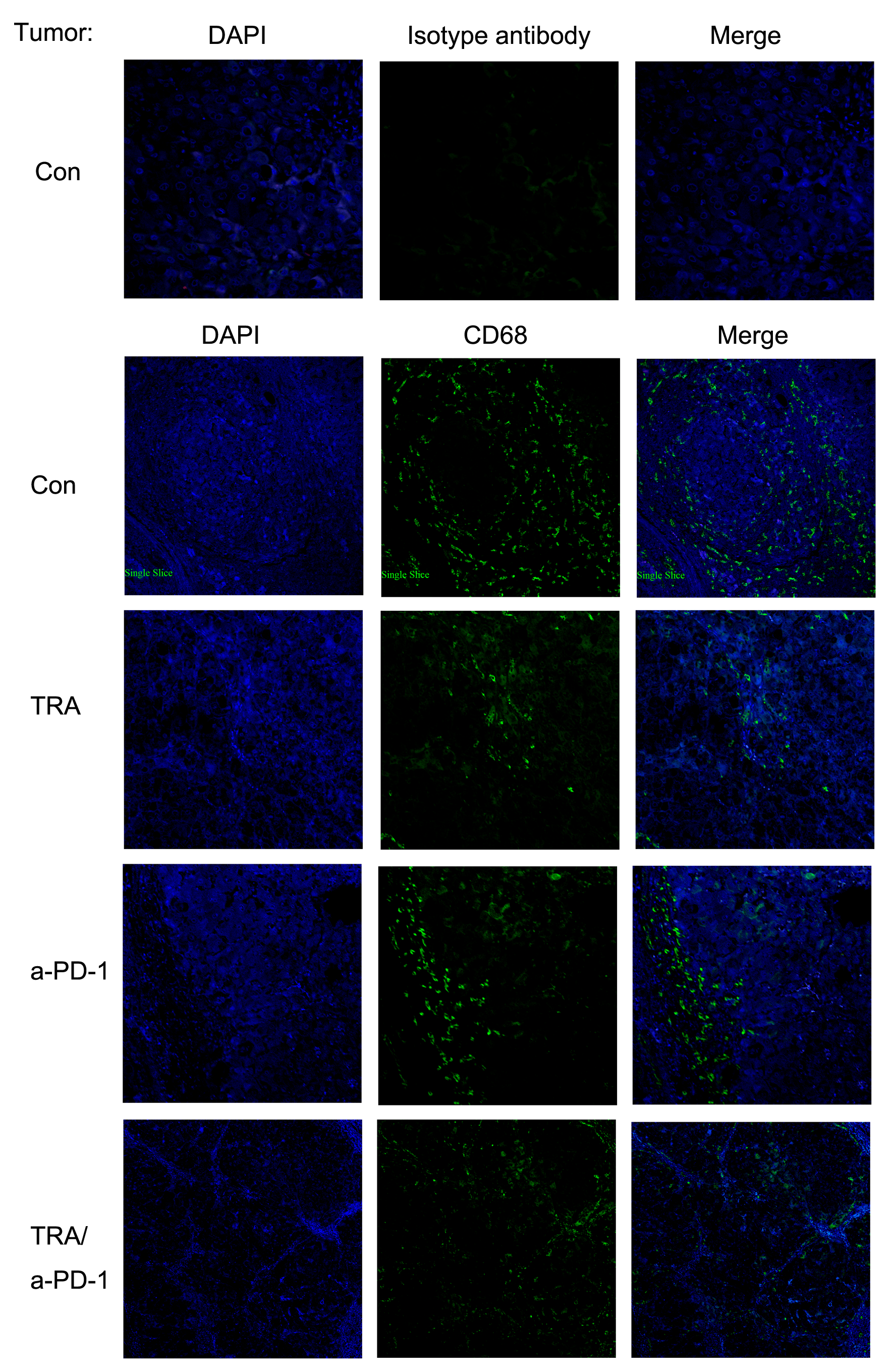

Supplement: Additional file 3: — Figure S2. The depleting effect of Trabectedin treatment on TAM within tumors. Mice were inoculated i.p. with 1 × 10^6^ ID8 cells and treated with either single or combined Trabectedin and α-PD-1 mAb as indicated in A on day 10 and 17. On day 19, tumor tissues harvested from treated mice were fixed with paraformaldehyde and blocked with 5% bovine serum albumin (BSA) for 45 min at room temperature. The samples were incubated with rat anti-mouse CD68 or rat IgG2a isotype control primary antibody at 4 °C for 16 h. An Alexa Fluor® 488-conjugated goat anti-rat IgG (H + L) polyclonal antibody was used as the secondary antibody. The samples were then analyzed for the presence of CD68-positive TAM by Olympus FV1000 confocal microscope system. The images are representative from one tumor in each treatment. [file 12967_2015_613_MOESM3_ESM.tiff]
